# RNA epitranscriptomics dysregulation: A major determinant for significantly increased risk of ASD pathogenesis

**DOI:** 10.3389/fnins.2023.1101422

**Published:** 2023-02-16

**Authors:** Athanasios Beopoulos, Manuel Géa, Alessio Fasano, François Iris

**Affiliations:** ^1^Bio-Modeling Systems, Tour CIT, Paris, France; ^2^Division of Pediatric Gastroenterology and Nutrition, Mucosal Immunology and Biology Research Center, Center for Celiac Research and Treatment, Massachusetts General Hospital for Children, Boston, MA, United States

**Keywords:** Autism spectrum disorder (ASD), maternal inflammation, mRNA alternative splicing, mRNA poly(A)-tail modulation, adenosine-to-inosine RNA editing, RNA epitranscriptomics

## Abstract

Autism spectrum disorders (ASDs) are perhaps the most severe, intractable and challenging child psychiatric disorders. They are complex, pervasive and highly heterogeneous and depend on multifactorial neurodevelopmental conditions. Although the pathogenesis of autism remains unclear, it revolves around altered neurodevelopmental patterns and their implications for brain function, although these cannot be specifically linked to symptoms. While these affect neuronal migration and connectivity, little is known about the processes that lead to the disruption of specific laminar excitatory and inhibitory cortical circuits, a key feature of ASD. It is evident that ASD has multiple underlying causes and this multigenic condition has been considered to also dependent on epigenetic effects, although the exact nature of the factors that could be involved remains unclear. However, besides the possibility for differential epigenetic markings directly affecting the relative expression levels of individual genes or groups of genes, there are at least three mRNA epitranscriptomic mechanisms, which function cooperatively and could, in association with both genotypes and environmental conditions, alter spatiotemporal proteins expression patterns during brain development, at both quantitative and qualitative levels, in a tissue-specific, and context-dependent manner. As we have already postulated, sudden changes in environmental conditions, such as those conferred by maternal inflammation/immune activation, influence RNA epitranscriptomic mechanisms, with the combination of these processes altering fetal brain development. Herein, we explore the postulate whereby, in ASD pathogenesis, RNA epitranscriptomics might take precedence over epigenetic modifications. RNA epitranscriptomics affects real-time differential expression of receptor and channel proteins isoforms, playing a prominent role in central nervous system (CNS) development and functions, but also RNAi which, in turn, impact the spatiotemporal expression of receptors, channels and regulatory proteins irrespective of isoforms. Slight dysregulations in few early components of brain development, could, depending upon their extent, snowball into a huge variety of pathological cerebral alterations a few years after birth. This may very well explain the enormous genetic, neuropathological and symptomatic heterogeneities that are systematically associated with ASD and psychiatric disorders at large.

## 1. Introduction

Among the psychiatric disorders that develop in children, Autism spectrum disorder (ASD) is perhaps the most severe, persistent and difficult to treat, mainly due to its multifactorial neurodevelopmental origin, which results in great heterogeneity across the spectrum of the condition (Faras et al., 2010). Diagnosis is based on behavioral observation, pointing to deficiencies in social interaction and communication, as well as constrained and repetitive behavioral patterns of activities and interests. As the name implies, there is inherent heterogeneity in ASD, with frequent psychiatric and medical comorbidities such as attention deficit/hyperactivity disorder, intellectual disability, anxiety disorder, and oppositional defiant disorder (Masi et al., 2017).

While the mechanisms underlying ASD remain poorly understood, they focus on alterations in CNS development and their implications for brain function (Watts, 2008). ASD is thus largely defined as a developmental disorder affecting neural connectivity, which affects the organization of the cortical network and the ratio of neural excitation to inhibition. However, little progress has been made toward the causes for laminar-specific excitatory and inhibitory cortical circuits disruption (Watts, 2008; Prem et al., 2020). Recent genetic studies have also identified that risk genes substantially converge in the development of the cortex between the 8 and 24th week of gestation (GW8-24) (Courchesne et al., 2019; Beopoulos et al., 2022). However, the majority of animal studies on ASD focus primarily on postnatal development and defects in synaptic transmission, rather than on early developmental processes guiding central nervous system (CNS) formation, such as cell differentiation, proliferation, migration, and arborization. Instead, it is assumed that the multigenic state of ASD is dependent on epigenetic effects, without however being able to pinpoint such factors (Eshraghi et al., 2018; Prem et al., 2020). Indeed, monozygotic (MZ) twins with different phenotypic traits exhibit considerable epigenetic variation as well as differentially methylated ASD-related gene loci (Kaminsky et al., 2009; Gordon et al., 2012).

Nevertheless, besides the possibility for differential epigenetic markings directly affecting the relative expression levels of individual genes or groups of genes, there are at least three mRNA epitranscriptomic mechanisms, which function cooperatively and could, in association with both genotypes and environmental conditions, alter spatiotemporal proteins expression patterns during brain development, at both quantitative and qualitative levels, in a tissue-specific, and context-dependent manner. These are differential mRNAs alternative splicing (Vuong et al., 2016; Stark et al., 2019), cytoplasmic-shortening or elongation of mRNAs poly(A)-tails (Eckmann et al., 2011; Weill et al., 2012) and adenosine-to-inosine (A-to-I) RNA editing (Slotkin and Nishikura, 2013; Liu et al., 2014) which addresses not only mRNAs but also RNA interference pathways (RNAi). All three mechanisms are known to function cooperatively and produce an enormous diversity of protein isoforms endowing the CNS with considerable phenotypic plasticity and functional adaptability (Gommans et al., 2009).

Here, we explore the postulate whereby, in the pathogenesis of ASD, RNA epitranscriptomics might probably take precedence over differential epigenetic (methylation/acetylation) modifications which require environmental conditions to stay stable long enough to have real physiological effects. Indeed, RNA epitranscriptomics affect not only real-time differential expression of receptor and channel proteins isoforms, playing a prominent role in CNS development and functions, but also similarly affect RNAi which, in turn, impact the expression of receptor and channel proteins irrespective of isoforms. This may very well explain the enormous genetic and symptomatic heterogeneities that are systematically associated with psychiatric disorders at large and not only with ASD.

## 2. Materials and methods

The authors conducted an in-depth review of the literature and used a systems biology approach to integrate the complex mechanisms of fetal brain development to then decipher the most probable origins of the neurodevelopmental alterations encountered in individuals with ASD. Throughout our literature review, we strongly favored studies on human subjects, and in the few instances where this was not possible, this is clearly stated in the text. The analytical procedure implemented (CADI™: Computer-Assisted Deductive Integration, BM-Systems, Paris, France) associates algorithmics and heuristics. The logic behind this model-building approach does not assume functional linearity within biological systems and the components of a model do not incorporate solely what is known. Indeed, since this approach relies upon strict and systematic implementation of negative selection of hypotheses, models arising from this procedure contain elements that have never been described but cannot be refuted by current knowledge and/or available biological data, thereby generating novel understanding. This model-building approach has proven its efficacy in a number of biological research domains, including the discovery of hitherto unsuspected biological mechanisms, pathways, and interactions directly associated with phenotypic transitions *in vivo* (be they pathological or developmental) (Gadal et al., 2003, 2005; Iris et al., 2009; Pouillot et al., 2010; Turck and Iris, 2011; Iris, 2012; Nussbaumer et al., 2016). CADI™ modeling has led to discoveries and patents in the fields of infectious diseases, oncology, neurology, psychiatry, dermatology, immunology, metabolic disorders, innovative bioprocesses for industrial biotech and the creation of new companies exploiting these patents. CADI™ models describe the biological phenomena involved in pathological states and provide novel mechanistic integrations to explain the cause of certain diseases, identify and select predictive biomarkers, and offer new combinations of molecules and new therapeutic strategies. Further information on the CADI method can be found in Iris et al. (2018).

## 3. Analysis

### 3.1. The genetic and epigenetics of increased risks in ASD pathogenesis

Genetic factors undeniably contribute to the risk for ASD (Abrahams and Geschwind, 2008). However, the genetics of autism are extremely complex and there are currently at least 1034 genes known to confer risk of ASD^1^, while as yet undefined environmental factors, in conjunction with genotype, play a prominent role in the etiology of ASD. Most of the 126 genes strongly predisposing to autism, in the context of a syndromic (e.g., fragile X syndrome) as well as idiopathic disorder, in addition to most of the 194 genes identified as significant predisposition candidates, bear rare single gene mutations, each of which, should they also be causal, would have a plethora of deleterious physiological consequences far beyond autism *per se*. Thus, genomic variants at risk for ASD may be rare, *de novo* and high effect variants, independently or in combination with common or inherited low effect variants (Gratten et al., 2014). However, previous studies have revealed that ASD-risk genes exhibit a huge range of spatiotemporal expression patterns in human brain (Ben-David and Shifman, 2013; Parikshak et al., 2013; Willsey et al., 2013; Chang et al., 2015) and only a small percentage of probands can be explained by identified ASD risk-genes (De Rubeis et al., 2014; Sanders et al., 2015). Indeed, sequencing of the whole genome of 85 families of ASD quartets (parents and two affected siblings) showed that while 42% of individuals with ASD had ASD-relevant mutations, only 31% of sibling pairs carried the same variants, highlighting the genetic heterogeneity occurring within families (Yuen et al., 2015).

This suggests that although the combination of specific interactions between different genes appears necessary to cause ASD, environmental factors, post-translational modifications (PTMs), and epigenetics should contribute to the manifestation of ASD-related specificities (Muhle et al., 2004). However, among the few studies that assessed epigenetic features in individuals with ASD, it appears that even if differential methylated variants at specific CpGs are identified, they have a very small-magnitude effect [< 10% absolute difference; reviewed in: (Siu and Weksberg, 2017; Waye and Cheng, 2018; Wiśniowiecka-Kowalnik and Nowakowska, 2019)].

In this context, it is important to note that environmental factors such as maternal psychological stress and prenatal infections (rubella, influenza, cytomegalovirus, etc.) influence epigenetic mechanisms, such as DNA and/or histones methylation, histones acetylation, and microRNA expression (Alegria-Torres et al., 2011; Barua and Junaid, 2015), which can then affect fetal endocrine programming and brain development (Babenko et al., 2015; Vaiserman, 2015). Hence, environmental factors act in a synergistic manner with genetic factors to influence inter-individual DNA methylation differences. Furthermore, these individual differences in methylation not only vary between cell types (Nardone et al., 2017) but are also not stable over time (Wong et al., 2010; Vogel Ciernia and LaSalle, 2016). It is worth to consider that chromatin regulatory genes are among high-risk variants associated with ASD (Walker et al., 2019). Changes in the chromatin environment can also alter RNA splicing mechanisms and this could be an indirect way in which mutations in chromatin regulatory genes could affect RNA epitranscriptomic mechanisms. This however, is unlikely to affect entire networks (Voineagu et al., 2011), so as to then lead to focal disorganization and altered minicolumn distribution typical to ASD cortex (Donovan and Basson, 2017).

Therefore, besides the possibility for differential epigenetic markings directly affecting the relative expression levels of individual genes or groups of genes, RNA epitranscriptomic mechanisms also could, in association with both genotypes and environmental conditions, alter spatiotemporal proteins expression patterns at both quantitative and qualitative levels, in a tissue-specific and context-dependent manner. Indeed, differential mRNAs alternative splicing, cytoplasmic-shortening or -elongation of mRNAs poly(A)-tails and adenosine-to-inosine (A- to -I) RNA editing, which are known to function cooperatively, alter gene expression and function in multiple ways. Among these, the amino acid (AA) sequence of proteins and their expression can be altered due to codon change during mRNA translation, pre-mRNA splicing, alteration of RNA stability by modification of nuclease recognition sites, or through changes in miRNA production and targeting, as well as alteration of RNA-protein interactions (Christofi and Zaravinos, 2019; Licht et al., 2019). About 3,817 differentially edited sites, addressing 943 target transcripts, have been identified in specific association with autism as compared to schizophrenia and genetic amyotrophic lateral sclerosis (Karagianni et al., 2022). Furthermore, 30–40% of brain-specific microexon splicing processes are found disrupted in over a third of ASD affected individuals (Irimia et al., 2014). The differentially edited transcripts are involved in molecular processes that include synaptic transmission, hypoxic conditions, endosome/lysosome function, cytoskeleton rearrangements, apoptosis, protein, and RNA processing.

### 3.2. The RNA epitranscriptomic mechanisms

#### 3.2.1. mRNA alternative splicing

The majority of mammalian multi-exon genes, through changes in splice site selection, generate multiple mRNA isoforms that give rise to proteins of different function and structure, or alter mRNA stability, translation, and localization. Exons and introns can be selected from the precursor mRNA and the translation product can give rise to isoforms with conflicting functions, or differential temporal and/or spatial expression patterns, resulting in considerable plasticity, and adaptability (Wang et al., 2015).

**FIGURE 1:**
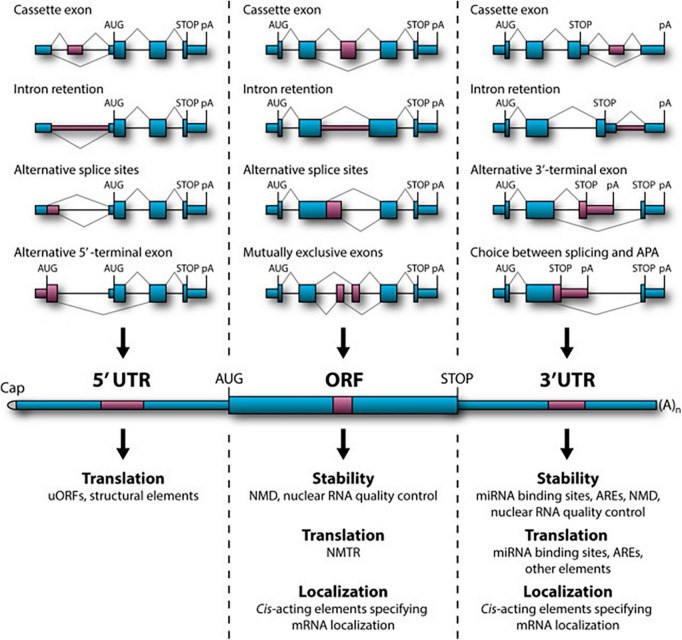
Role of alternative splicing (AS) in mRNA stability, translational activity and subcellular localization. **(Top)** Examples of relevant AS topologies; mid: mature mRNA containing 5′ and 3′UTRs flanking the protein-coding ORF; **(bottom)** downstream regulation outcomes of AS events in the corresponding regions. APA, alternative cleavage and polyadenylation; ARE, AU-rich element; NMD, nonsense-mediated decay; NMTR, nonsense-mediated translational repression; uORF, upstream open reading frame (ORF). Figure obtained from Mockenhaupt and Makeyev (2015) under a creative commons license 3 and 4.

Nearly 95% of human gene transcripts are subject to alternative splicing (AS) (Pan Q. et al., 2008; Wang et al., 2008; Johnson et al., 2009). Splicing regulation is extensively used in the mammalian nervous system to generate specific protein isoforms that influence every aspect of neurodevelopment and function (Zheng and Black, 2013; Raj and Blencowe, 2015) with splicing defects being strongly associated with neurological and neurodegenerative diseases. Recent genome-wide studies of AS in mice have emphasized its occurrence throughout development within several regions of the nervous system (Dillman et al., 2013; Yan et al., 2015). In particular, AS is involved in cell fate decisions during cortical neurogenesis (Linares et al., 2015; Zhang et al., 2016), synaptogenesis and synaptic plasticity [reviewed in Raj and Blencowe (2015) and Vuong et al. (2016)]. AS events are considerably regulated by *cis*-regulatory RNA sequences and *trans*-acting splicing factors (Wang and Burge, 2008; Kornblihtt et al., 2013). Numerous AS topologies have been documented, comprising interchange among alternative 5′ or 3′ splice sites, alternative 5′ or 3′ terminal exons (A5E and A3E), cassette exons, mutually exclusive exons, intron retention (IR), and a range of arrangements among these options (Wang and Burge, 2008). For most groups of splicing events, several splicing sites will be present concomitantly on the nascent pre-mRNA. In addition, a large proportion of AS events take place in the 5′ and 3′ untranslated regions (UTR) of the mRNA bordering the open reading frame (ORF). Even if this has no effect on the polypeptide sequence, it can modulate other features of the mRNA performance. Furthermore, many AS-mediated alterations in the ORF sequence have been shown to affect mRNA stability, subcellular localization and translational activity (Figure 1; Braunschweig et al., 2013; Hamid and Makeyev, 2014; Sibley, 2014). AS should therefore not be seen as a binary “splice or not” event, determined by the intrinsic properties of the splice site in question. Rather, it is a context-specific competition in which the splicing machinery must discriminate and choose between multiple splice sites.

**FIGURE 2:**
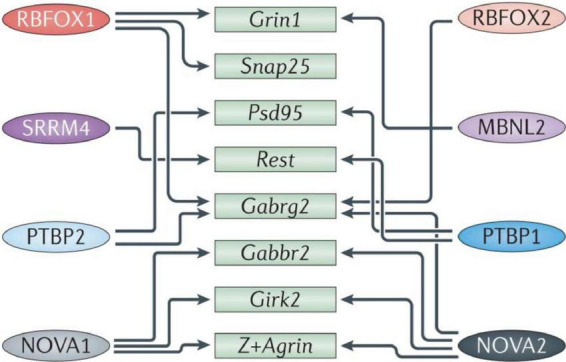
Diagram representing some of the splicing mechanisms in the brain. Numerous transcripts important in brain and neurological development (green boxes) are cross-regulated (indicated by arrows) by multiple RNA-binding proteins (RBPs; colored ovals on the **left** and **right**). Girk2, inwardly rectifying potassium channel Kir3.2; Gabrg2, GABAA receptor subunit gamma 2; Gabbr2, GABAB receptor 2; MBNL, muscleblind-like; NOVA, neuro-oncological ventral antigen; Psd95, postsynaptic density protein 95; PTBP, polypyrimidine tract binding protein; Rest, repressor element 1-silencing transcription factor; RBFOX, RNA-binding protein fox 1 homolog; Snap25, synaptosome-associated protein 25; SRRM4, serine/arginine repetitive matrix protein 4. Figure from Vuong et al. (2016) obtained by Springer Nature under license No 5430810425823.

Specialized pre-mRNA binding proteins control alternative splicing patterns by altering spliceosome assembly at specific splice locations [for review, see (Fu and Ares, 2014; Matera and Wang, 2014; Lee and Rio, 2015)]. These proteins differ in structure and depending on their binding position, interactions with cofactors and signaling pathway modification, can have a variety of impacts on a target transcript. While certain splicing regulators are found throughout the brain, others are only found in certain tissues. The hindbrain and ventral spinal cord express NOVA1, while the forebrain and dorsal spinal cord express NOVA2, with considerable overlap in the midbrain and hindbrain (Yang et al., 1998). In addition, different neuronal cell types exhibit unique maturation susceptibility through the expression of different combinations of regulators [reviewed in Li et al. (2007)] which also affect different, often overlapping, alternative splicing programs, as each transcript is typically targeted by several regulators (Figure 2).

As cells move along the neural lineage, their alternative splicing patterns vary substantially. They are induced by modification in the expression of certain RNA-binding proteins, such as the polypyrimidine tract binding proteins 1 and 2 (PTBP1, 2) and serine/arginine repetitive matrix protein 4 (SRRM4; known as nSR100). PTBP1 depletion from cultured fibroblasts is found to induce their *trans*-differentiation into neurons (Xue et al., 2013), whereas PTB1 loss in mice mutants causes precocious neurogenesis and depletion of neural stem cells, diminishing the population of ependymal cells that ascend from radial glia. Mice depleted for PTBP2 resulted in altered stem cell positioning and proliferation (Poduri et al., 2013). Furthermore, loss of splicing regulators like NOVA2 and RNA-binding protein fox-1 homolog 2 (RBFOX2), which regulate alternative splicing of components of the Reelin signaling system, cause abnormalities in cortical, and cerebellar lamination (Yano et al., 2010). For instance, mis-splicing of the Reelin component disabled 1 (Dab1) causes multiple types of layer II/III and IV neurons to migrate improperly (Poduri et al., 2013).

**FIGURE 3:**
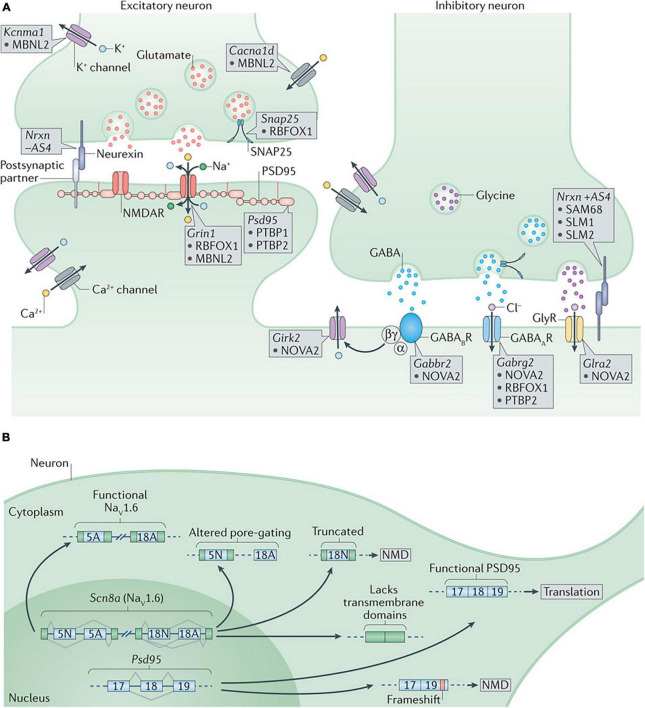
**(A)** Alternative splicing regulation of synaptogenesis and synaptic function. At the presynaptic terminal, alternative splicing of synaptosomal-associated protein 25 (Snap25) by RNA-binding protein fox 1 homolog 1 (RBFOX1/A2BP1) and of the calcium-activated potassium channel subunit alpha 1 (Kcnma1) by muscleblind-like 2 (MBNL2) are important to control neurotransmitter release. Differential splicing of the presynaptic neurexins (Nrxns) at AS4 by KHDRBS proteins (SAM68, SLM1, and SLM2) controls targeting to postsynaptic partners. At excitatory synapses, alternative splicing of the transcript encoding the NMDA receptor subunit GluN1, Grin1, is regulated by RBFOX1 (A2BP1) and MBNL2, whereas the polypyrimidine tract binding proteins (PTBPs) control productive splicing of the scaffold protein, postsynaptic density protein 95 (Psd95). Splicing of the transcripts encoding L-type voltage-gated calcium channels, such as the pore-forming subunit Cav1.3 (encoded by Cacna1d), by MBNL2 may allow the voltage sensitivity, conductance, or other properties to be tuned as synapses differentiate. At inhibitory synapses, neuro-oncological ventral antigen 2 (NOVA2) mediates alternative splicing of the transcripts encoding many postsynaptic components such as the metabotropic GABAB receptor (Gabbr2), the inwardly rectifying potassium channel Kir3.2 (Girk2) and the glycine receptor alpha 2 (Glra2). Splicing of the GABAA receptor subunit transcript (Gabrg2) is controlled by multiple splicing regulators including NOVA2, RBFOX1 (A2BP1) and PTBP2. **(B)** Alternative splicing regulation of synaptogenesis and synaptic function. Alternative splicing controls the expression and function of many synaptic components. Expression of PSD95 is repressed by PTBP-controlled exclusion of exon 18 until late in neuronal maturation when it is required for synaptogenesis. The gene encoding the voltage-gated sodium channel Nav1.6, Scn8a, has multiple alternative exons (such as 5N, 5A, 18N, and 18A as shown in the figure) that can change its gating properties, determine its localization or alter its overall function. GABAAR, GABAA receptor; GABABR, GABAB receptor; GlyR, glycine receptor; NMD, nonsense-mediated decay; NMDAR, NMDA receptor; SAM68, SRC-associated in mitosis 68 kDa protein. Figure from Vuong et al. (2016) obtained by Springer Nature under license No 5430810425823.

Many aspects of synaptic function are also influenced by alternative splicing factors, including synapse specificity *via* the KH domain-containing, RNA-binding, signal transduction-associated (KHDRBS) family, regulation of inhibitory synapses *via* NOVA2, and splicing of synaptic components and ion channels and *via* Muscleblind-like 2 (MBNL2), the sodium channel modifier 1 (SCNM1), and the neuronal ELAV-like (nELAVL) proteins (SCNM1) RBFOX1 and RBFOX2 (Vuong et al., 2016; Figure 3).

One of the alternative splicing targets of KHDRBS, are the neurexins (Nrxn), encoding presynaptic cell surface proteins that form a heterophilic adhesion complex with neuroligins at neuronal synapses. They promote synaptogenesis through *trans*-synaptic signaling by organizing pre- and postsynaptic compartments in a bidirectional manner. The synapse-specific functions of neurexins variants are conferred through extensive alternative splicing and controlled by various genes and promoters (Craig and Kang, 2007). To give an order of magnitude, pre-mRNAs from the three Nrxn genes produce more than 2,000 protein isoforms by alternative splicing (Baudouin and Scheiffele, 2010). Mutations in neuroligin and neurexin genes are associated with neurodevelopmental disorders, including ASD (Wang et al., 2018).

The role of neurexins in the regulation of synaptogenesis is reminiscent of an adhesive program that depends on the affinity of the generated variants for post-synaptic, cell type-specific ligands. To illustrate, alternative splicing at exon 20 generates the NRX4 + protein variants with a 30 AA insertion (Ex4 +), whereas omission of exon 20 results in the NRX4- variant (ΔEx4). Here, the inclusion of exon 20 is negatively regulated by KHDRBS (Craig and Kang, 2007). The NRX Ex4 + and ΔEx4 variants show differential interactions with neuroligins, leucine-rich repeat proteins (LRRTMs), and the Cbln1-GluD2 complex that are critical mediators of synaptogenesis (Uemura et al., 2010). ΔEx4 isoforms are found to preferentially bind neuroligin 1B that is abundantly found at glutamatergic synapses, whereas Ex4 + isoforms favorably bind neuroligin-2 (NLGN2A) at GABAergic and glycinergic synapses (Chih et al., 2006).

Additionally, alternative splicing is both spatially and temporally controlled. In mice, neuronal activity induces changes in Nrxn1 splice isoform selection through calcium/calmodulin-dependent kinase IV (CAMKIV) signaling. The KH-domain RNA-binding protein SAM68 associates with RNA response elements in the Nrxn1 pre-RNA and regulates the incorporation of exon 20, and this also varies between brain regions. In the developing cortex, Nrxn ΔEx4 mRNAs decrease in accordance with the developmental evolution (post-natal days 0–21) of granule cell’s resting potential (−25 to −55 mV) (Rossi et al., 1998). In parallel, depolarization results in an activity-dependent change between Ex4 (+) and ΔEx4 mRNAs, increasing the expression of the latter by t10-fold, while decreasing Ex4 (+) variants and total Nrxn1 transcripts. This is suggestive of a depolarization-induced change (and thus cell type specific) in the turnover of pre-existing Nrx1 mRNA (Iijima et al., 2011). Dysfunction of SAM68 binding results in motor coordination deficits in mice (Iijima et al., 2011).

**FIGURE 4:**
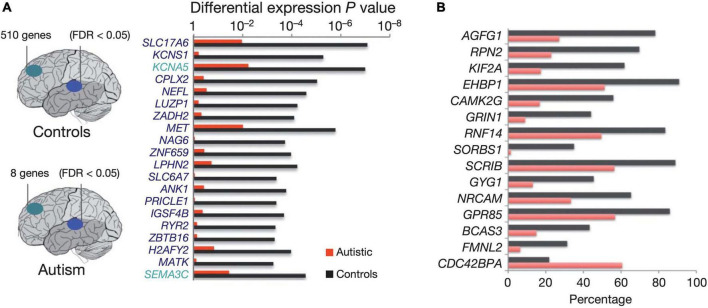
Diagram depicting the number of genes showing significant expression differences between frontal and temporal cortex in control samples (top) and autism samples (bottom). **(A)** Top 20 genes differentially expressed between frontal and temporal cortex in control samples. All of the genes shown are also differentially expressed between frontal and temporal cortex in fetal mid-gestation brain, but show no significant expression differences between frontal and temporal cortex in autism. The horizontal bars depict *p* values for differential expression between frontal and temporal cortex in the autism and control groups. **(B)** Top A2BP1 (RBFOX1) -specific differential splicing (DS) events. DS events showing the most significant differences in alternative splicing between low-A2BP1 autism cases and controls as well as DS differences consistent with the A2BP1 binding site position. The horizontal axis depicts the percentage of transcripts including the alternative exon. Red-autism samples, black-control samples Figure modified from Voineagu et al. (2011) obtained by Springer Nature under license No 5430811097703 and 5431300363740.

Another consequence of altered early developmental programs specific to ASD is the deregulation of layer formation and layer-specific neuronal migration in prenatal cerebral cortex development (Beopoulos et al., 2022). Moreover, the gene expression profiles that, in neurotypical development (ND), distinguish the frontal from the temporal cortex are significantly attenuated (Johnson et al., 2009), implying that transcriptional and splicing dysregulation are among the underlying mechanisms of neuronal dysfunction in this disorder. Furthermore, in ASD brains, lower cognitive functioning is associated with higher densities of apical pyramidal cell dendrites along layer II and within the cortical lobe of layer IV (Hutsler and Zhang, 2010). Concomitantly, ASD brains display considerably dysregulated splicing of the ataxin-2 binding protein 1 A2BP1 (RBFOX1)-dependent alternative exons (Voineagu et al., 2011; Figure 4).

Concurrently, while nuclear A2BP1 regulates the alternative splicing of a variety of neuronal transcripts, it is itself alternatively spliced to produce cytoplasmic isoforms that regulate mRNA stability and translation. Cytoplasmic Rbfox1 binds to 3′-UTR of mRNA targets, increasing their abundance and in particular those associated with cortical development and autism (Lee et al., 2016). CLIP-seq from ASD-like mice brains show enrichment in Rbfox1 targets involved in the regulation of brain development and in particular in the control of synaptic activity and calcium signaling (Weyn-Vanhentenryck et al., 2014). In parallel, Rbfox1 nuclear downregulation is found to alter neuronal positioning during corticogenesis (embryonic day 14.5) in mice. The defects were found to occur during radial migration and terminal translocation (Hamada et al., 2016). Furthermore, A2BP1, along with MBNL2 which also mediates pre-mRNA alternative splicing regulation, controls the splicing of GRIN1 (NMDA-type ionotropic glutamate receptor subunit 1 -NMDAR1) (Vuong et al., 2016). GRIN1 exon five skipping prolongs the time course of excitatory NMDAR postsynaptic currents (EPSCs) at thalamic relay synapses and increases spine density along apical dendrites of pyramidal neurons in layer V (Liu et al., 2019).

However, A2BP1 (RBFOX1) is not the only alternative splicing factor dysregulated in human ASD brains. SRRM4 (nSR100) is only expressed in the CNS (Calarco et al., 2009), and is found downregulated in autism (Irimia et al., 2014). SRRM4 alters neuronal destiny and differentiation (Makeyev et al., 2007; Raj et al., 2011) by promoting alternative splicing and inclusion of neural-specific exons in target mRNAs (Calarco et al., 2009). Depletion of SRRM4 in mice results in abnormal cortical lamination by halting neurogenesis of upper-layer neurons and causing accumulation of progenitor cells in lower-layer neurons (Poduri et al., 2013). The most characteristic class of nSR100-regulated exons are microexons, which are largely conserved, frame-preserving, neurally enriched cassette exons ranging from 3 to 27 bp in length (Irimia et al., 2014). Microexons are significantly enriched in genes with crucial involvement in synaptic biology and genetic links to ASD, and they typically encode residues of surface of proteins, affecting protein-protein interactions (Irimia et al., 2014; Li et al., 2015). Microexon processing-related splicing defects are associated with ASD. Comparisons between large cohorts of autism and control brain samples showed disrupted splicing of 30–40% brain-specific microexons in over a third of individuals with ASD (Irimia et al., 2014).

#### 3.2.2. mRNA-specific poly(A)-tail modulation

Following gene transcription in eukaryotic cells, the newly formed mRNA matures by processing of both ends. Nuclear polyadenylation of the 3′ end by poly-A polymerase adds a poly(A) tail to the mRNA molecule, contributing to the translational regulation of the transcript by increasing its stability and allowing its export from the nucleus [reviewed in Di Giammartino et al. (2011)]. With the exception of replication-dependent histone mRNAs, that instead of a poly(A) tail end in a histone downstream element (a stem-loop structure followed by a purine-rich sequence), all other mRNAs acquire around 250–300 adenosine residues (Siianova et al., 1990; Marzluff et al., 2002). However, the exact 3′ polyadenylation position is tightly regulated and defines the regulatory susceptibility of the mature transcript. In addition, the exact length of the poly(A) tail is also closely regulated in both nucleus and the cytoplasm and defines the transport, translation and recycling rate of the mature transcripts [reviewed in Zhang et al. (2010), Eckmann et al. (2011), and Weill et al. (2012)]. When the mRNA reaches the cytoplasm, the poly(A) tail, along with the 5′ cap, stabilize the closed loop that is formed by the attachment of the cap structure to the translation initiation factor 4F (eIF4F) complex, and thus contribute to the initiation of the translation process (Kahvejian et al., 2005). The structure and integrity of the newly circularized mRNA becomes the target of non-mRNA-specific translation regulatory elements (Dever, 2002; Beilharz et al., 2010), while transcript-specific translational control is made possible by *cis*-acting regulatory sequences (Abaza and Gebauer, 2008). These regulatory elements typically reside in the 3′UTR, although they can be also present in the 5′ UTR or the coding sequence, and assemble mRNA specific ribonucleoprotein complexes (mRNPs), deadenylation mRNPs and cytoplasmic polyadenylation complexes that dynamically modulate the length of the poly(A) tail (Piqué et al., 2008; Chekulaeva et al., 2011), which, in turn, defines the extent of mRNA translation (Beilharz and Preiss, 2007). Silent mRNPs localize and accumulate in specific cell regions to be reactivated by cytoplasmic poly(A) elongation when and where the encoded proteins are needed. The acquisition of the poly(A) tail and the subsequent modification of its length, can be thus seen as a dynamic process that regulates gene expression in time and space. In general, ASD cortices, as shown by post mortem studies, present a global poly(A)-tail shortening pattern that remarkably impacts several high confidence ASD-risk genes (Parras et al., 2018).

To give an illustrative example, alternative polyadenylation of the serotonin transporter SLC6A4/SERT1 leads to symptoms of anxiety and depression with impaired retention of fear extinction memory, mimicking the phenotype of SERT1 KO mice (Battersby et al., 1999; Gyawali et al., 2010). The mouse SLC6A4 gene yields two AS products with 123 bp differences that lead to a polyadenylation polymorphism. The human gene contains a single T/G nucleotide polymorphism (rs3813034) in the more distal of the two polyadenylation signals, influencing the forms of polyadenylation that occur in the brain (Battersby et al., 1999; Gyawali et al., 2010). In both mice and humans, impaired retention of fear extinction memory is selectively impaired in homozygous carriers of the G allele (Wellman et al., 2007; Narayanan et al., 2011), which is associated with a lower distal polyadenylated fraction, as the T allele results in greater utilization of the polyadenylation site. Functionally, the distal polyadenylation sequence element is positively correlated with the equilibrium level of SERT1 mRNA (Gyawali et al., 2010) and is located next to a microRNA (miR-16) binding site (Baudry et al., 2010), implying that binding of regulatory proteins to the distal sequence element could regulate miR-16 binding (Shanmugam et al., 2008) and modify SIRT1 translation (Baudry et al., 2010; Hartley et al., 2012). This establishes a clear link between SERT1-mediated activity and the use of polyadenylation in the amygdala-prefrontal cortex network. The distal polyadenylated fraction of SERT1 in brain tissue of men is significantly higher than that of women, independent of rs3813034 genotype. Panic disorder is a female-predominant condition, with an approximately twofold higher prevalence in women than in men. Therefore, the G allele of SERT1 rs3813034 appears to be the risk allele in women (Gyawali et al., 2010). Developmental hyperserotonin (DHS) is the most consistent neurochemical finding reported in autism and has been implicated in the pathophysiology of ASD (Hough and Segal, 2016).

**FIGURE 5:**
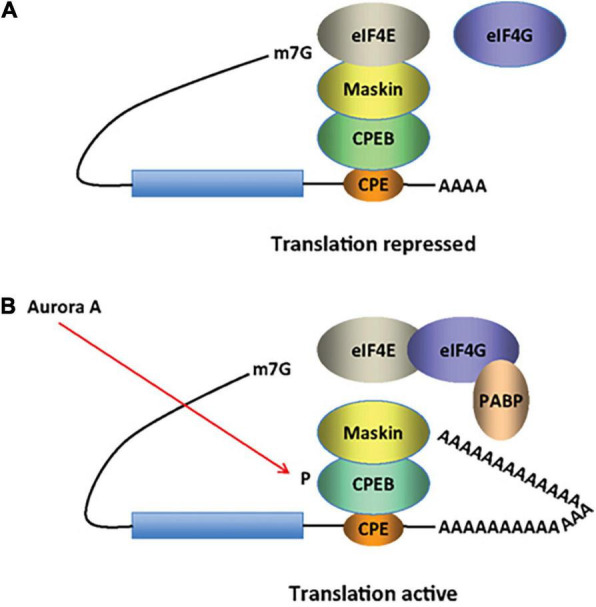
CPEB activity in inducing cytoplasmic shortening or elongation of poly(A)-tails. Some mRNAs contain a cytoplasmic polyadenylation element (CPE), which is bound by CPEB. **(A)** CPEB also interacts with Maskin (or Neuroguidin, a functionally related protein), which in turn interacts with the cap (m7G) binding protein eIF4E. Such mRNAs are translationally inactive because Maskin inhibits the association of eIF4E and eIF4G, another initiation factor that helps recruit the 40S ribosomal subunit to the 5’ end of the mRNA. **(B)** Cues such as NMDA receptor activation stimulate the kinase Aurora A, which phosphorylates CPEB, an event that causes poly(A) tail elongation. poly(A) binding protein (PABP) binds the newly elongated poly(A) tail and recruits eIF4G. The PABP-eIF4G dimer helps to displace Maskin from eIF4E, allowing eIF4G to bind eIF4E and initiate translation [figure modified from Zukin et al. (2009), obtained under a creative commons license 3 and 4].

Furthermore, with respect to ASD susceptibility, cytoplasmic polyadenylation element-binding proteins 1-4 (CPEB1-4) bind to mRNAs containing CPE sequences, and activate or repress translation by inducing cytoplasmic elongation or shortening of their poly(A) tails (Ivshina et al., 2014). In the developing brain, and in response to environmental embryonic cues, CPEBs regulate mRNA transcription of cell communication mediators, such as hormones, that are involved in memory formation and learning, through modulation of synaptic plasticity (Si et al., 2003; Sarkissian et al., 2004; Ivshina et al., 2014; Fioriti et al., 2015). Synaptic plasticity is further achieved by activity-dependent local translation of dendritic mRNAs. In early mice development, CPEB resides at synaptic sites of hippocampal neurons. Polyadenylation at synapses is mediated by the N-methyl-D-aspartate receptor (NMDAR)-related activation of Aurora, one of the kinases that phosphorylates CPEB (Huang et al., 2002; Figure 5). In the mammalian adult brain, CPEB is localized in postsynaptic densities of the dendritic layers of the hippocampus (Wu et al., 1998). While CPEB KO mice exhibit deficits in synaptic plasticity and memory formation, FMR1/CPEB1 double-knockout (KO) rescues the fragile X-like phenotype of FMR1-KO mice (Zukin et al., 2009). The fragile X syndrome (FXS) is the most common-known genetic cause of autism and is characterized by the loss of fragile X mental retardation 1 protein (FMRP).

**FIGURE 6:**
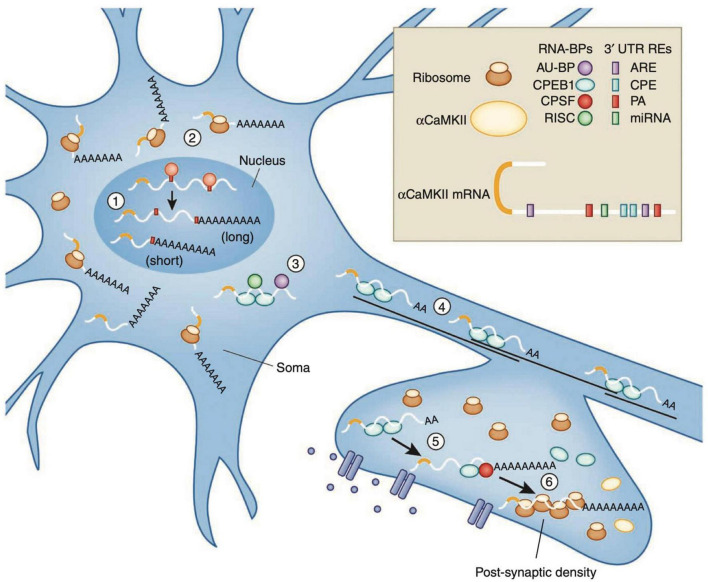
Recycling of αCaMKII mRNA in neurons. Alternative polyadenylation (APA) of αCaMKII mRNA generates two transcripts with 3′ UTRs of different lengths (1). Both transcripts are polyadenylated in the nucleus, but the composition of the *cis*-elements in their 3′ UTR modifies their cytoplasmic fates. The short isoform is translated mainly in the soma (2), whereas miRNA binding sites, AU-rich elements (AREs) and cytoplasmic polyadenylation elements (CPEs) mediate deadenylation, repression (3) and transport (4) of the long transcript to dendrites. Upon neuronal stimulation in the post-synaptic densities (PSD), CPEB1 promotes αCaMKII mRNA polyadenylation (5), and αCAMKII local translation (6). Figure from Weill et al. (2012), obtained by Springer Nature under license No 5430811375131.

Memory consolidation uses a similar mechanism (Figure 6): αCaMKII, which is also found at the dendrites of hippocampal neurons, contains CPEs in its 3′ UTR, and in the rat brain is polyadenylated by CPEB1 when stimulated by light (Wu et al., 1998). In addition, αCaMKII phosphorylates and activates CPEB1 (Atkins et al., 2005; Weill et al., 2012), creating a positive feedback loop that stabilizes synaptic inputs (Aslam et al., 2009). Mice expressing CamKII lacking the 3′UTR, have reduced protein levels, severely affecting late-phase long-term potentiation, spatial and long-term memory (Miller et al., 2002).

Comparison of transcript levels from the cortices of 43 post-mortem idiopathic ASDs and 63 NT controls showed no change in CPEB1 and 2 levels, whereas CPEB3 and CPEB4 were found slightly decreased and increased, respectively. However, at the protein level, CPEB4 was significantly decreased, obeying the ASD-specific trend of poly(A) tail shortening, and showed an isoform imbalance due to a reduced inclusion of the neuron-specific micro-exon 4 (ΔEx4 > Ex4 +) (Theis et al., 2003; Parras et al., 2018). This micro-exon encodes the eight amino acid “‘B region” that inserts motifs for PTMs, such as phosphorylation by S6K, AKT, PKA, or PKC (Theis et al., 2003). Of note, most transcripts of high-risk ASD genes are linked by CPEB4 and over 40 of them, including Dyrk1a, FOXP1, and WAC SFARI database (see text footnote 1), show reduced poly(A) tails as well as reduced protein levels. When mice are exposed to an equivalent imbalance of CPEB isoforms, their levels of CPEB transcript-dependent proteins are reduced and they exhibit ASD-like behavioral, neuroanatomical, and electrophysiological phenotypes (Parras et al., 2018). Another epitranscriptomic mechanism, with major implications for brain function across lifespan, including early brain development, is adenosine-to-inosine RNA editing, the effects of which may contribute to the development of the neuroanatomical and behavioral characteristics of ASD.

#### 3.2.3. Adenosine-to-inosine RNA editing

Eukaryotes use RNA editing, where an RNA molecule is chemically modified at the nucleotide base, as a posttranscriptional mechanism that greatly increases transcript diversity from a limited size genome. In the CNS, the predominant form of RNA editing is the deamination of adenosine (A) to form inosine (I), that is then recognized as guanosine (G) during translation. A-to-I editing is biologically significant in both translated and UTR of RNA, with consequences ranging from trivial to critical, affecting molecular stability, gene expression and function. When editing occurs in the coding sequence, it is referred to as a “recoding event” which can also alter splice sites and base pairing, thus affecting the secondary structure of the RNA. It further changes the binding specificities with other molecules such as mi/siRNAs and proteins. The results of most editing events are still unknown (Yang et al., 2021), with recent research suggesting that the majority of pre-mRNAs are edited (Franzen et al., 2018).

**FIGURE 7:**
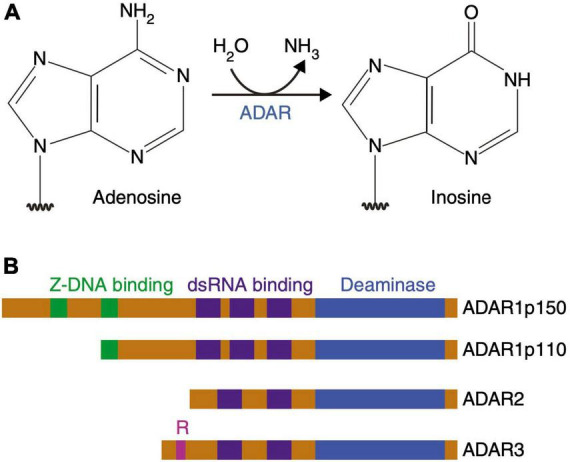
Adenosine deamination and the ADAR enzyme family. **(A)** ADAR enzymes catalyze the A-to-I hydrolytic deamination reaction, by which an adenosine loses an amine group and is converted to inosine. **(B)** There are four main proteins of the ADAR enzyme family: two isoforms of ADAR1 (p110 and p150), ADAR2 and ADAR3. All of these enzymes contain a conserved deaminase domain, shown in blue. The double-stranded (ds) RNA-binding domains, shown in purple, determine substrate specificity. The two ADAR1 isoforms differ in their Z-DNA-binding domains, shown in green. ADAR3 contains an arginine-rich domain, shown in pink, which binds single-stranded RNA. Figure from Slotkin and Nishikura (2013), obtained by Springer Nature under license No 5431360984026.

The reaction is carried out by the “Adenosine Deaminases that Act on RNA” enzyme family (ADAR 1, 2, and 3 enzymes in vertebrates) (Bass, 2002; Slotkin and Nishikura, 2013). ADAR1 and ADAR2 are expressed throughout the body, although they are more prevalent in the CNS (Palladino et al., 2000; Nishikura, 2010), whereas ADAR3 localizes exclusively in the brain and is considered catalytically inactive. According to current knowledge, ADAR1 is associated with hyper-editing, being the major editor of repetitive sites, ADAR2 with site-selective editing, being the major editor of non-repetitive coding sites, while ADAR3 may act as an inhibitor of editing by competing with ADAR1 and 2 for RNA binding (Tan et al., 2017). ADAR1 is ubiquitously expressed from two different promoters, one being constitutively active, giving rise to the nuclear p110 isoform (cADAR1), and the other being interferon induced (p150), resulting in a version of the ADAR1 enzyme (iADAR1) that is partially localized to the cytoplasm (Veno et al., 2012; Figure 7).

The extent of RNA editing by ADAR proteins is vast, with millions of sites already identified in the human genome (Tariq and Jantsch, 2012; Bazak et al., 2014; Picardi et al., 2017). It has major implications for nervous system function throughout life (Savva et al., 2012), and a review of A-to-I editing rates in the brains of six different age groups (fetus, infant, child, adolescent, middle-aged, and elderly) revealed 748 sites with significant differences in editing rates between all groups, with 742 of these sites showing an increasing pattern of editing during development from fetal to adult samples. This increase in the A-to-I editing pattern is associated with cortical layer growth and neuronal maturation (Hwang et al., 2016).

**FIGURE 8:**
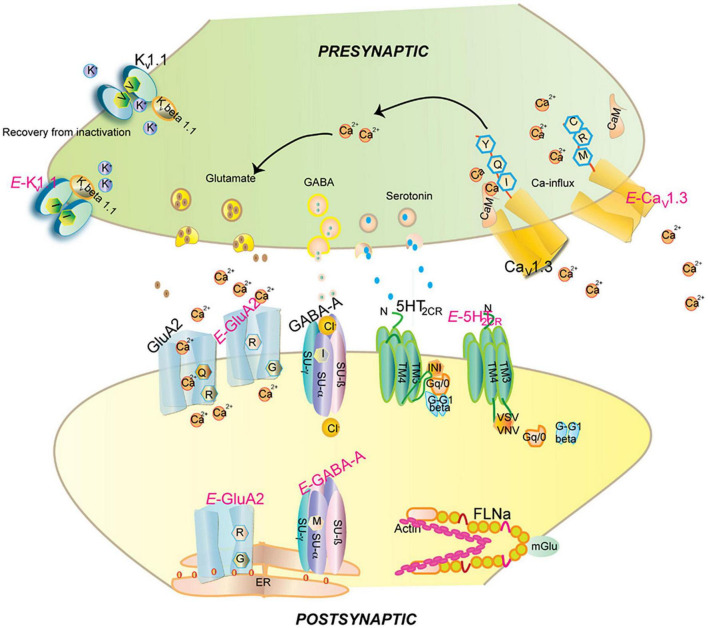
Impact of editing on selected neuronal receptors and proteins. Shown are several receptors and channels in their unedited (labeled in black) and edited (E, labeled in pink) versions. Editing at the Q/R site of ionotropic glutamate receptor GRIA2 (GluA2) subunit decreases Ca2 + permeability and endoplasmic reticulum exit efficiency. Membrane trafficking of the GABA_A_ receptor is reduced by editing of I to M in the alpha3 subunit. Editing of the serotonin 5-HT2c receptor converts the amino acids I-N-I to V-S-V, V-G-V, or V-N-V. This reduces G-protein coupling in the receptor. The editing-induced I to V exchange in the potassium voltage-gated channel KCNA1 (Kv1.1) alters the interaction with KCNB1. Editing of the IQ motif in voltage-gated calcium channel CACNA1D (Cav1.3) to MR abolishes calmodulin binding. Filamin-α (FLNA) is edited in a region that is known to interact with metabotropic glutamate receptor GRM7 (mGlu7) and some of its relatives. Figure from Tariq and Jantsch (2012), obtained under a creative commons license 3 and 4.

Proteins derived from edited pre-mRNAs vary considerably in function and most often affect receptors and ion channels widely expressed in the brain. In addition, proteins involved in cytoskeletal remodeling, thereby contributing to neuronal growth and plasticity, are strongly affected by RNA editing. In general, editing of protein-coding RNAs leads to the generation of protein isoforms and diversification of protein functions (Tariq and Jantsch, 2012; Figure 8).

A global bias for A-to-I hypo-editing in ASD brains, with the number of downregulated RNA editing sites across brain regions in ASD far exceeding those that were upregulated, involving many synaptic genes, has recently been reported. The fragile-X protein, FMRP, interacts with ADAR1 and ADAR2 in an RNA-independent manner, whereas FXR1P interacts only with ADAR1. FXR1 and FMR1 showed a negative (inhibitory) and positive (enhancing) correlation with termination changes, respectively. Furthermore, there is a converging pattern of RNA editing alterations in ASD and fragile X syndrome, providing a molecular bridge between these related disorders (Tran et al., 2019).

##### 3.2.3.1. Brain proteins significantly affected by adenosine-to-inosine mRNA editing

###### 3.2.3.1.1. Glutamate-gated ion channels

Five subunits of the ionotropic glutamate ion channel (AMKA) GRIA (GluA2, GluA3, GluA4, GluK1, and GluK2) undergo RNA editing in their coding regions by ADAR (Bass, 2002). Four editing sites where an AA substitution occurs have been identified, specifically arginine to glycine (R/G), glutamine to arginine (Q/R), tyrosine to cysteine (Y/C), and isoleucine to valine (I/V). In GluA2 (GRIA2), the Q/R substitution (CAG to CIG) plays a critical role in the function of the ion channel, as it is located in the pore loop domain, rendering the channel impermeable to Ca^2+^. GluA2 subunits are edited in almost 100% of all transcripts at the Q/R site (Daniel et al., 2017). The consequences of (sub) editing are critical, as ADAR2 KO homozygous mice die within weeks of birth due to repetitive seizures caused by excessive Ca^2+^ influx. However, the mice can be rescued by introducing the GRIA2*^R^* mutation that carries the Q/R substitution (Higuchi et al., 2000; Greger et al., 2006).

In GluA2 and 3, the c-terminal domain binds to the PDZ domains 4–6 of the neuronal scaffolding proteins GRIP1 and 2 (glutamate receptor interacting proteins 1 and 2; 7 PDZ domains in total). Loss of Grip expression in mice results in delayed recycling of GluA2 and increases sociability. Adversely, gain-of-function mutations in Grip proteins (GRIP1 to PDZ4-6), observed in ASD patients contribute to reduced social interactions (Han et al., 2017).

Both GRIP1 and GRIP2 (glutamate receptor-interacting proteins) exist in different splice isoforms, with GRIP1b/2b isoforms including an alternative 18 amino acid domain, excluded form GRIP1a/2a, at their extreme N-terminus, that determines the palmitoylation fate of the protein. For instance, GRIP1a (non-palmitoylated) and GRIP1b (palmitoylated) splice variants inhibit and increase NMDA-induced AMPAR internalization in cultured hippocampal neurons respectively (Hanley and Henley, 2010). Hence, up- regulated GRIP1/2 alternative splicing, due to decreased mRNA A-to-I editing, increases GRIP1a/2a isoforms availability, and could promote decreased sociability and social interactions.

However, AMPA receptors are associated with a large network of proteins (at least 14 members), many of which have multiple alternatively spliced isoforms, that play important roles in receptor membrane trafficking, by guiding the receptor from the cell body to the synapse and by possibly regulating the downstream signal transduction pathway (Song and Huganir, 2002; Santos et al., 2009). In addition, AMPAR subunits are subject to numerous PTMs that give rise to various combinatorial effects. For example, GluA1 undergoes 11 PTMs that can occur independently and reversibly (seven phosphorylations, two palmitoylations, one ubiquitination, and one S-nitrosylation), resulting in 2^11^ potential GluA1 variants, each of which is expected to exhibit slightly altered properties. This number will be further increased by considering the heteromeric nature of AMPARs (Diering and Huganir, 2018).

###### 3.2.3.1.2. GABA_A_ receptors

The ligand gated chloride channel receptors GABA_A_ are comprised of five subunits: 2 α sub-units, 2 β subunits, and either a γ or a δ subunit (Hevers and Luddens, 1998). Additionally, there exist 6 α, 3 β, 3 γ, and 4 δ sub-units allowing for even greater generation and assembly diversification of stoichiometries. Furthermore, the α3 subunit can be edited at position 343, resulting in an isoleucine (I: AUA) to methionine (M: AUI) codon change (Ohlson et al., 2007; Tian et al., 2011). The I/M change results in delayed currents and faster inhibition upon GABA stimulation (Rula et al., 2008), apparently due to decreased subunit stability and overall receptor trafficking, probably by altering α and γ subunits, or ligand, interaction, thereby reducing its cell surface expression (Daniel et al., 2011; Tariq and Jantsch, 2012). Editing of the α3 subunit is developmentally regulated, with its pre-mRNA going from unedited at embryonic day 15 to over 80% edited at postnatal day 7 (Ohlson et al., 2007; Rula et al., 2008). The expression of the unedited α3 GABA receptor during development is crucial for synapse formation (Ben-Ari et al., 2007) and its overall expression decreases with age concomitantly with the increase of the expression of the α1 subunit (Hutcheon et al., 2004).

Molecular genetics and pharmacological data designate α1-containing GABA_A_ receptors as “sedative” subtype(s) and α2 and/or α3-containing receptors as “anxiolytic” subtype(s) while agonists of the α5-containing receptors, such as diazepam, impair cognition (Atack, 2011a,b). systemic decrease in GABA_A_R signaling, reduced sociability and attention, while blocking cortical GABA_A_R impaired social behavior and attention. Since the effects of specific-receptor ligands are marginal, α5-containing GABA_A_ receptors could be involved in attention deficits but do not appear to contribute to reduced sociability (Paine et al., 2020), thus potentially increasing the weight of α3 subtype hypo-editing on ASD-related social behavior.

###### 3.2.3.1.3. Serotonin 2C receptor

Similarly, the serotonin 2C receptor (5-HT2CR) is subject to combinatorial editing at five sites [referred to as A, B, C’ (E), C, and D sites] found within the G protein coupling domain (second intracellular loop), which plays a key role in mammalian cerebral cortex development and is targeted by the only FDA-approved drugs to treat autistic symptoms, risperidone and aripiprazole.

Cortical circuit development is a complex process that involves the differentiation, proliferation, and migration of epithelial, glial, and neural cell subtypes, followed by synapse formation and curation of redundant connections through synaptic pruning. While these processes are largely controlled by genetic programming, they are further regulated by a wide range of extrinsic signals (such as hormones, growth factors, guidance signals, cell adhesion molecules, etc.) as well as environmental influences. Serotonin (5-HT) is a key regulator of neural circuit development by modulating neuronal proliferation, migration and differentiation. Most of its effects are mediated by the 14 5-HT receptors, all of which are G protein-coupled, with the exception of the ionotropic 5-HT type 3A receptor (5-HT3A) which is responsible for rapid neuronal activation (Takumi et al., 2020; Beopoulos et al., 2022). Dysregulation of the 5-HT system is involved in the development of several neuropsychiatric disorders, including intellectual disability, autism, depression and anxiety (Kinast et al., 2013; Brummelte et al., 2017; Velasquez et al., 2017).

**FIGURE 9:**
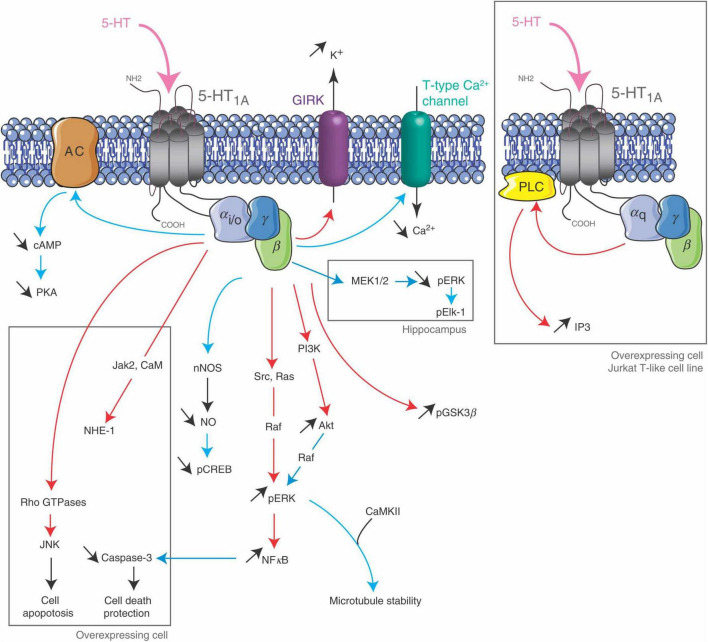
5-HT2C receptor signaling pathways. The 5-HT2CR is coupled to PLC in neurons and choroid plexus, and its activation leads to an accumulation of IP3. It inhibits K + conductance in both neurons and choroid plexus and stimulates an apical Cl- conductance in choroid plexus. It may also be coupled to PLD in transfected cells and choroid plexus. Figure from Masson et al. (2012), obtained under a creative commons license 3 and 4.

In mammals, during early cortical development and up to 16 weeks of gestation (GW16), 5-HT is of maternal and/or placental origin (Cote et al., 2007; Bonnin and Levitt, 2011; Beopoulos et al., 2022), whereas later, serotonergic afferents invade the cortex and the developing fetus initiates its own 5-HT production (Gaspar et al., 2003; Vitalis and Rossier, 2011; Budday et al., 2015; Nowakowski et al., 2016; Beopoulos et al., 2022). Importantly, 5-HT signaling is influenced by numerous epigenetic and genetic factors, as well as by perinatal stress (Papaioannou et al., 2002; Provenzi et al., 2016), infection and inflammation (Winter et al., 2009; Gupta et al., 2015), 5-HT metabolism and storage, (Popa et al., 2008) and genetic alterations (Pluess et al., 2010; Karg et al., 2011). 5-HT2CRs are known for their prevalent role in anxiety, with their editing converting the amino acids INI to VSV, VGV, or VNV. Mice possessing only the fully edited VGV isoform of 5-HT2CR, which thereby overexpress brain 5-HT2CR, notably at isoforms diversity and neurotrophic expression levels, are strikingly predisposed to post-traumatic stress-like disorder (PTSD), a trauma- and stress-related disorder characterized by dysregulated fear responses and neurobiological impairments (Martin et al., 2013; Regue et al., 2019; Figure 9).

Editing of the five sites in the G protein-coupling domain of 5-HT2CR changes three codons [AAU (asparagine), AUA (isoleucine) and AUU (isoleucine), i.e., 157-IRNPI-161 at I, N and I] (Bezanilla, 2004), leading to a possible six different 5-HT2CR isoforms. Overall, up to 24 receptor isoforms are expressed with considerably modified G protein–coupling functions, impacting 5-HT effectiveness and ligand-binding affinity (Nishikura, 2010). Extensively edited receptor isoforms, as the fully edited VGV, activate G protein less efficiently than non-edited (i.e., INI) receptors (Schmauss, 2003).

Furthermore, although studies in mice have shown that 5-HT2C pre-mRNA editing is regulated in a 5HT-dependent manner, the efficacies of the hallucinogenic drug lysergic acid diethylamide (LSD) and other serotonergic drugs are also regulated by RNA editing. This suggests that modification of the editing efficiencies or patterns generates populations of 5-HT2CRs that respond differentially to serotonergic drugs (Niswender et al., 2001), while simultaneously RNA editing efficiency is also influenced by the drugs themselves (Gurevich et al., 2002). Taken together, the data from transgenic mice strongly indicate that the editing status of the 5-HT2CRs can directly influence behavior, highlighting the significance of RNA editing in the etiology and development of psychiatric disorders.

In the human prefrontal cortex (PFC), the most abundant 5-HT2C mRNA sequences originate from the editing of the A site, or from the editing arrangements AC’C, ABCD, and ABD. While there are no appreciable differences in the 5-HT2CR RNA editing efficiencies in the prefrontal cortex of schizophrenia or major depressive disorder patients compared to controls, the editing pattern of 5-HT2CR mRNA is significantly different in the prefrontal cortex of depressed suicide victims (Niswender et al., 2001; Gurevich et al., 2002; Schmauss, 2003). Here, C’ site editing is considerably amplified, D site editing is significantly reduced, and the C site shows a tendency toward increased editing. When mice were treated with the antidepressant drug fluoxetine (Prozac) changes in C’, C, and D site editing were induced, that were the exact opposite of those observed in suicide victims (Gurevich et al., 2002). The comparison of relative isoform frequencies between ASD individuals and controls revealed considerable differences, with the most significant in MNV, IDV, and ISI isoforms, all under-represented in ASD brains (Eran et al., 2013).

###### 3.2.3.1.4. Voltage-gated calcium channels

The core sequence of the calmodulin-binding IQ domain of the of the pore-forming α1 subunit (CACNA1D) of the low voltage activated (LVA) L-type calcium channels Cav1.3 is also subject to A-to-I editing, although only in the brain (Huang et al., 2012). LVA channels are involved in multiple process such as neurotransmitter secretion, synaptic transmission, neuronal pace-making and modulation of other ion channels (Singh et al., 2008). LVA are Ca^2+^ influx channels and are subject to voltage depended inhibition (VDI) and Ca^2+^ depended inhibition (CDI).

The α1 subunit that forms the pore is composed by four domains (I–IV), and each domain has six transmembrane regions (S1–S6) (Catterall et al., 2005), with S1-S4 constituting the voltage sensitive domain and S5- S6 forming the central pore. S6 covers and blocks the inner surface of the pore. Upon activation, the S4 segment moves outward displacing the S6 domain and opens the pore (Swartz, 2004). Calmodulin (CaM) binds to C-terminus of the α1 subunit through its IQ domain with the Ca2 + -CaM complex formation resulting in CDI (Tadross et al., 2008). Ca^2+^ binding to the N- and C-terminal CaM lobes/hotspots, further regulates channel function (Dick et al., 2008).

**FIGURE 10:**
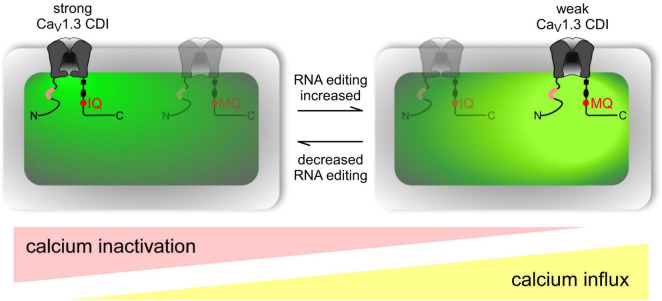
Overview of RNA editing effects on CaV1.3 Ca2 + -dependent inactivation (CDI) and calcium load in neurons. Channel schematic as defined in Figure above. Increased RNA editing **(right scenario)** favors channels with MQ and other edited versions of the IQ domain, decreasing overall CDI and presumably increasing cellular Ca2 + load (intense yellow-green coloration). Decreased editing **(left scenario)** favors default channels with IQ version of IQ domain, increasing overall CDI and potentially decreasing cellular Ca2 + load (weak yellow-green coloration). Actual neurons reside on a continuum between these two extremes, as represented by ramp schematics at bottom. Figure from Huang et al. (2012), obtained by Elsevier under license No 5430820583951.

The IQ domain of CACNA1D includes the ADAR-2 editable IQDY amino acid sequence. Editing generates multiple isoforms such as MQDY, MQDC, IRDY, MRDC, MRDY, and IQDC. The MQ and IR edits display weakened CDI, while MR edits show up to 50% reduced CDI with faster recovery upon inactivation. Reduced CDI increases repetitive action potentials and calcium spikes (Figure 10). Editing patterns are developmentally regulated, being absent in mice before embryonic day 14 (E14) while becoming prominent after postnatal day 4 (P4). Editing is spatially distributed along the brain, with the highest editing patterns found at the frontal cortex and hippocampus (Huang et al., 2012).

As noted above, edited CaV1.3s exhibit reduced CDI, and in neurotypical development, neurons in the suprachiasmatic nucleus display particularly reduced CDI, along with higher repetitive action potential frequencies, and calcium spike activity (Huang et al., 2012). The suprachiasmatic nucleus is the seat of the brain’s circadian clock (Gillette and Tischkau, 1999) and autism is frequently associated with co-morbidities such as disturbed sleep patterns and altered circadian rhythms which, in turn often result in impaired vigilance, learning, and memory abilities, and abnormal anxiety responses (Won et al., 2017; Zuculo et al., 2017).

Genetic studies have pointed to alterations in voltage-gated calcium channels as ASD-specific candidates, with mutations in most of the pore-forming and auxiliary subunits being present in autistic individuals (Breitenkamp et al., 2015). The most prominent among them, are those affecting the loci encoding the α subunits such as CACNA1D, CACNA1A, CACNA1B, CACNA1C, CACNA1G, CACNA1E, CACNA1F, CACNA1H, and CACNA1I along with those affecting their accessory subunits CACNA2D3, CACNB2, and CACNA2D4 (Liao and Li, 2020).

###### 3.2.3.1.5. Voltage-gated potassium channels

Voltage-gated potassium Kv1.1 neuronal channels are consisted of a pore forming tetramer of α-subunits, together with 4 β-subunits and accessory subunits. Through the opening and closing of the potassium selective pore, they regulate action potential and neuronal excitability. The human Kv1.1 (KCNA1) gene lacks introns and is susceptible to A-to-I RNA editing, resulting in an exchange of isoleucine for valine at the sixth transmembrane segment (S6, amino acid 400), positioned within the ion conducting pore (Bhalla et al., 2004). Kv1.1 channels in medulla, thalamus and spinal cord are edited up to 65–80% (Decher et al., 2010).

**FIGURE 11:**
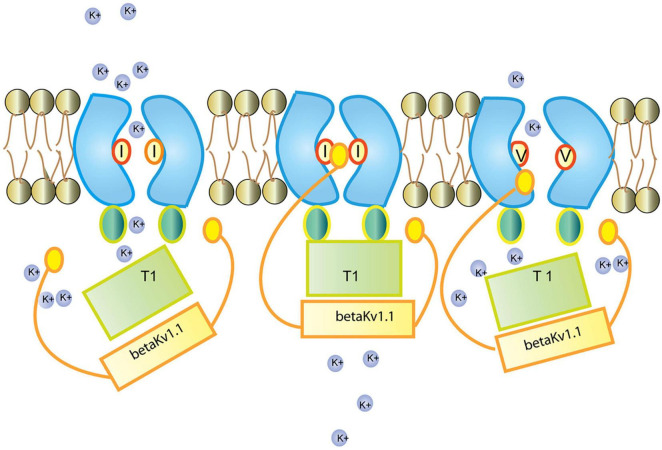
Regulation of the Kv1.1 (KCNA1) channel by Kvb1.1 (KCNE5). Kvb1.1 has an inactivation gate which interacts with the unedited (I) form of the Kv1.1 receptor. Editing of Kv1.1 changes the isoleucine to valine (V). This reduces the affinity for Kvb1.1 and enhances recovery from inactivation. While all Kv channels (voltage-gated) function along the same principles (Grizel et al., 2014), different Kv channel subtypes show very different responses to short stimulation (Cameron et al., 2017). Figure from Tariq and Jantsch (2012), obtained under a creative commons license 3 and 4.

In the ER, Kv1.1 associates with the redox sensor Kvb1 (Pan Y. et al., 2008). Kvb1 contains an inactivation domain at its N-terminal region, which regulates Kv1.1’s lag time and inactivation. In comparison to the unedited form of the channel, the edited Kv1.1 recovers from Kvb1-mediated inactivation 20 times faster (Bezanilla, 2004; Bhalla et al., 2004; Figure 11). It has been further demonstrated that while the Kv channel blocker 4-aminopyridine (4-AP) causes epileptic seizures in the unedited channels, the edited channels are rendered insensitive to 4-AP by severing the connection between the pore lining and the channel blocker (Streit et al., 2011). Arachidonic acid has been shown to have a similar lack of sensitivity toward edited channels (Decher et al., 2010).

###### 3.2.3.1.6. Filamins and actin organization

Homo- and heterodimers of FLNA and FLNB filamin proteins mediate orthogonal branching of actin filaments (Sheen et al., 2002; Popowicz et al., 2006), thereby participating in actin reorganization and vesicular trafficking, a process essential for, among others, cell motility, migration, dendritic spine, and synapse formation (Dillon and Goda, 2005; Popowicz et al., 2006). FLNA or FLNB depletion leads to defects in cardiovascular and bone development, with FLNA depletion ultimately causing embryonic death (Feng et al., 2006; Hart et al., 2006; Zhou et al., 2007).

Both FLNA and FLNB are subject to developmentally regulated editing that leads to glutamine (Q) to arginine (R) codon exchange in the highly conserved repeat 22 (Li et al., 2009; Wahlstedt et al., 2009), which is part of the 21–24 repeats, involved in the interaction with multiple proteins (Enz, 2002; Popowicz et al., 2006). Editing regulates the interaction of FLNA with glutamate receptors GRIA5a, 5b, 7b, 8a, and GRIA4a and 7a through their C-terminus. It also regulates its interaction with β-integrins and migfilin, an adaptor protein present at cell-cell and cell-extracellular matrix adhesion sites, linking the cytoskeleton to filamin. FLNA repeat 21 interacts with β-integrins, only if repeat 20 disassociates from it, with the efficiency of the association depending on the interactions between repeats 21–24 (Lad et al., 2007, 2008). Editing thus affects competition between different filamin ligands for common binding sites on FLN repeat domains with consequences for cell-cell interactions and signaling. Two β-integrins (ITGB3 and ITGB7) as well as at least one integrins-binding partner (CIB2), involved in the regulation of Ca^2+^-dependent mechano-transduction, are amongst the genes clearly associated with increased ASD risks in the SFARI Gene database (see text footnote 1).

The potassium channel KCND2 (Kv4.2) and FLNA also interact at filopodial roots, and FLNA is expressed in both cortical and hippocampus neurons. This interaction, which once more involves FLNA repeats 21–24, depends on a “PTPP” amino acid motif in Kv4.2 (AA601-604). Co-expression of filamin in heterologous cells through correct introduction of functional KV4.2 channels at the cell surface, increases the entire cell current density by around 2.7-fold (Petrecca et al., 2000).

Hence, FLNA editing is likely to change neuronal receptors and channels organization as well as synaptic transmission by altering the interaction profiles with binding partners. Furthermore, miRNAs are also the object of A-to-I RNA editing which, in turn, impacts the spatiotemporal expression of receptors, channels and regulatory proteins irrespective of isoforms.

##### 3.2.3.2. Brain-specific miRNAs subject to adenosine-to-inosine RNA editing

A probable key evolutionary function of A-to-I RNA editing might be the modulation of RNA interference (RNAi) efficacy, and this by competing for shared double-stranded RNA (dsRNA) substrates with the microRNA/small interfering RNA (miRNA/siRNA) pathway (Yang et al., 2005; Nishikura, 2006).

RNA interference (RNAi) is the specific suppression of gene expression by means of miRNA and/or siRNA. The siRNA inhibits the expression of a specific target mRNA with the process being initiated by the enzyme Dicer, which cleaves long dsRNA molecules into short dsRNAs. Following RNA separation into single strands, the sense strand will be cleaved by the protein Ago2 and incorporated into the structure of the RNA-induced silencing complex (RISC), which can now bind complementarily to its target mRNA and degrade it. miRNAs are non-coding genomic or exogenous RNAs that are instead involved in the regulation of several mRNAs. miRNAs are derived from a long RNA coding sequence (pri-miRNA) that is processed in the cell nucleus by the RNaseIII Drosha, aided by the dsRNA-binding protein DGCR8, to generate a 70-nucleotide stem-loop structure (pre-miRNA). The dsRNA portion of the pre-miRNA then undergoes the same downstream processing as the siRNA (initiated by Dicer) to form the mature miRNA molecule that can be incorporated into the RISC complex.

Dicer appears to discriminate between dsRNAs with I-U wobble base pairs and those with only Watson-Crick base pairs. The synthesis of siRNAs and miRNAs by Dicer is progressively decreased as ADARs deaminate dsRNAs. In fact, Dicer cannot cleave *in vitro* dsRNAs that have been significantly edited (50% of adenosines changed to inosines) by ADARs (Zamore et al., 2000; Scadden and Smith, 2001; Yang et al., 2005). Therefore, even minimal siRNA/miRNA editing can affect both mRNA targeting and miRNA/siRNA efficiency. miRNAs are found to be deregulated as a whole in patients with ASD, with no specific miRNAs being systematically altered among individuals (Abu-Elneel et al., 2008; Anitha and Thanseem, 2015).

**FIGURE 12:**
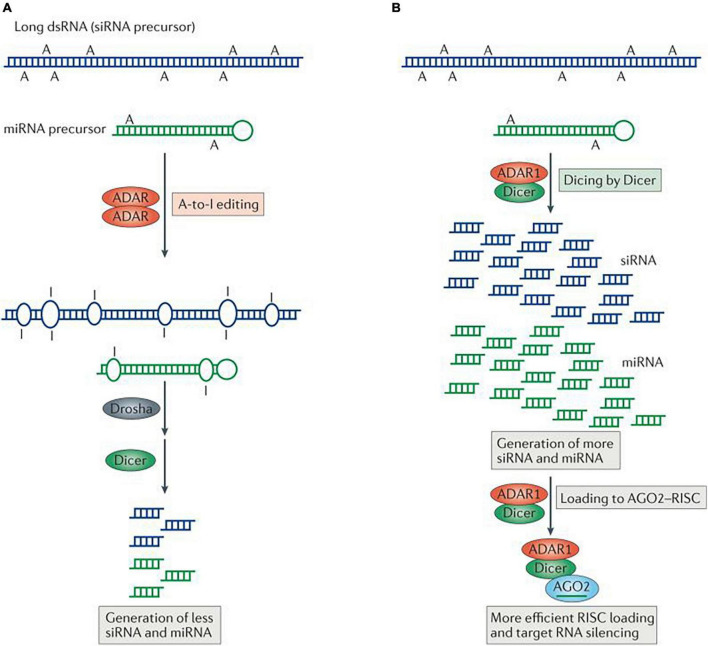
Regulation of RNA interference (RNAi) by adenosine deaminases acting on RNA (ADARs). Two different types of interaction between RNA-editing and RNAi pathways are known, one antagonistic and the other stimulative. **(A)** In antagonistic interactions, ADAR–ADAR homodimers edit long double-stranded RNA (dsRNA) and certain microRNA (miRNA) precursors. Editing changes the dsRNA structure and makes it less accessible to Drosha and/or Dicer, consequently decreasing the efficacy of RNAi by reducing the production of short interfering RNAs (siRNAs) and miRNAs. **(B)** In the case of stimulative interactions, ADAR1, as part of a Dicer–ADAR1 heterodimer, promotes RNAi by increasing the Dicer cleavage reaction rate, thereby generating more siRNAs and miRNAs and enhancing RISC (RNA-induced silencing complex) loading and target mRNA silencing. AGO2, Argonaute 2. Figure from Nishikura (2016), obtained by Springer Nature under license No 5430820144326.

Additionally, adenosine residues in UAG triplets may be modified more frequently, according to a wide analysis of previously established pri-miRNA editing sites. Among 209 pri-miRNAs containing UAG triplets, 43 UAG editing sites and 43 non-UAG editing sites in 47 pri-miRNAs were highly modified in the human brain. *In vitro* miRNA processing by recombinant Drosha-DGCR8 and Dicer-TRBP (TRBP: the RNA-binding cofactor of Dicer) complexes showed that the majority of pri-miRNA editing interferes with miRNA processing steps (Figure 12) and that editing generated new types of miRNAs. miRNA editing could have a significant impact on RNAi gene silencing, as approximately 16% of human pri-miRNAs are subject to A to I deamination (Kawahara et al., 2008).

**FIGURE 13:**
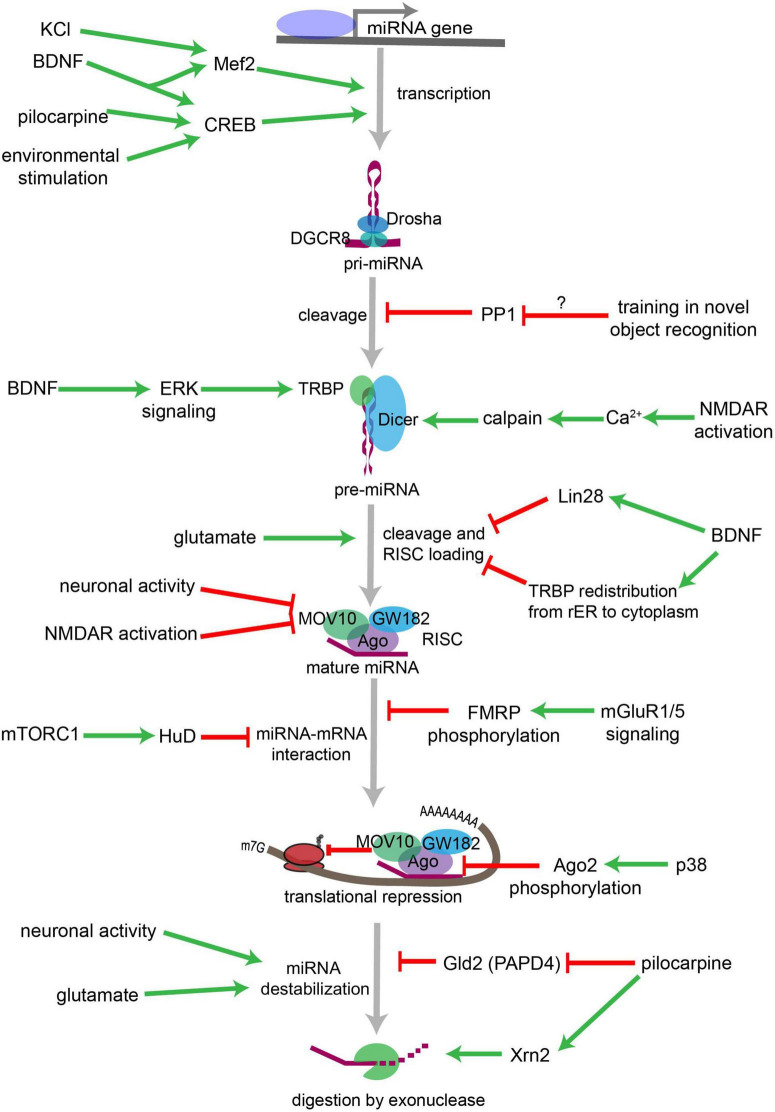
Overview of neuronal signaling events that influence miRNA biogenesis, activity, and degradation. Each step within the miRNA biogenesis pathway may be stimulated (green arrow) or inhibited (red bar) by intra- and extracellular signaling events. Pri-miRNA levels increase when BDNF or other signals activate transcription factors that stimulate the transcription of miRNA-encoding genes. Pri-miRNA cleavage by the Microprocessor is influenced by the activity of proteins, such as PP1 which inhibits Microprocessor activity. Pre-miRNA cleavage by Dicer is increased in response to glutamate, BDNF signaling, or NMDA receptor activation. BDNF can also inhibit Dicer’s ability to cleave some pre-miRNAs by inducing Lin28 binding to the pre-miRNA terminal loop or by promoting TRBP redistribution and dissociation from Dicer. Neuronal activity and NMDA receptor activation inhibit RISC activity by promoting the degradation of the RISC component Mov10. miRNA interactions with target mRNAs are also influenced by RNA binding proteins such as FMRP or HuD neuron-specific HuD and HuB are subject to adenosine-to-inosine RNA editing at five editing sites each (Enstero et al., 2010), which are regulated by mGluRs (GRMs) and mTORC1 signaling, respectively. P38-induced phosphorylation of Ago2 stimulates mRNA translation by causing the RISC to release its bound miRNA. miRNAs may also be destabilized by increases in neuronal activity, by glutamatergic signaling, or by pilocarpine-induced inhibition of the miRNA stabilizing protein Gld2. Pilocarpine may also stimulate miRNA degradation by increasing expression of the exonuclease Xrn2. Figure from Thomas et al. (2018), obtained under a creative commons license 3 and 4.

In the brain, CNS signaling events alter miRNA levels and activities, adjusting the response of individual neurons to changing cellular contexts (Figure 13). Conversely, miRNAs control the synthesis of proteins that govern synaptic transmission and other types of neuronal signaling, which in turn shapes neuronal communication. Regulation of axonal and dendritic growth, spine development and synaptogenesis by a number of miRNAs has been shown to be critical to brain development. During early development, miRNAs principally foster neuronal differentiation, whereas at later stages, they primarily serve as molecular brakes throughout synaptic development and plasticity (Bicker et al., 2014). Neurological deficits in humans have been associated with defects in miRNA production, and significant changes in miRNA levels occur in traumatic brain injury, epilepsy and in response to less serious brain insults in rodent models (Thomas et al., 2018). The representation and development of some of these diseases in mouse models can also be modified by manipulating specific miRNAs (Thomas et al., 2018).

In cultures of young primary hippocampal neurons at different developmental stages (stage 2–4), the expression of most neuronal miRNAs remains low during early development but increases consistently over the course of neuronal differentiation. A specific subset of 14 miRNAs displayed decreased expression at stage 3, and sustained expression when axonal growth was observed. In mature hippocampal neurons after 21 days of culture, 210 miRNAs were expressed, with 114 at high levels. Following induction of neuronal activity, 51 miRNAs, comprising miR-134, miR-146, miR-181, miR-185, miR- 191, and miR-200a demonstrated altered expression patterns after NMDA receptor-dependent plasticity, and 31 miRNAs, including miR-107, miR-134, miR-470, and miR-546 were upregulated by homeostatic plasticity protocols (van Spronsen et al., 2013).

**FIGURE 14:**
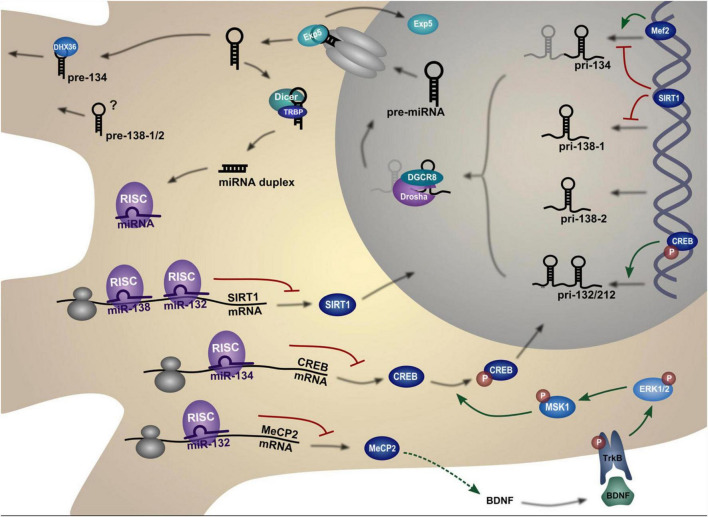
Biogenesis and regulation pathways of dendritic miRNAs. The miRNA gene is transcribed into a primary miRNA transcript (pri-mRNA) which is cleaved by Drosha to generate a hairpin miRNA precursor (pre-miRNA). After nuclear export, the pre-miRNA is cleaved by Dicer to form the double-stranded miRNA duplex. One strand of this duplex, the mature miRNA, is then incorporated into the miRNA-induced silencing complex (miRISC). The miRISC complex can bind to complementary target mRNAs, thereby repressing their translation. The figure depicts those targets that are in turn regulating miRNA expression in neurons. Figure from Bicker et al. (2014), obtained by Springer Nature under license No 5430820320321.

The miR-134 gene is a negative regulator of dendritic spine size. miR-134 localizes to the postsynaptic compartment in cultured neurons and controls translation of the kinase Limk1, which is essential for the development of dendritic spines, by phosphorylating and inactivating the actin depolymerizing factor Cofilin. This causes a reorganization of the actin cytoskeleton. Dendritic spine size is decreased by miR-134 overexpression, whereas Limk1 restoration rescues spine morphology (Schratt et al., 2006). Therefore, miR-134 regulates neuronal excitability and dendritic spine morphology by preventing Limk1 mRNA from being translated, which restricts the size of dendritic spines (Jimenez-Mateos et al., 2015; Figure 14).

Recent studies have linked ASD, intellectual disability, and schizophrenia to microdeletions encompassing MIR137HG, the host gene that encodes miR-137 part of the miR-379-410 cluster, which includes 39 miRNAs, located in the DLK1-GTL2 paternally imprinted locus in mice (DLK1-DIO3 region in humans). The miR-137 appears to regulate many types of synaptic plasticity as well as signaling cascades that are thought to be abnormal in schizophrenia, acting upstream in many important neurodevelopmental pathways. In addition, miR-137, which has over 1300 predicted targets, regulates adult neurogenesis, synaptic plasticity, and glutamatergic signaling (Pacheco et al., 2019).

**FIGURE 15:**
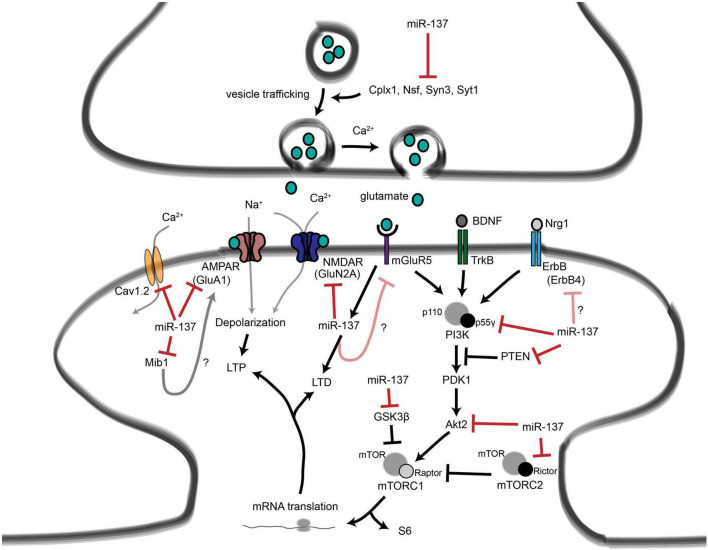
Roles for miR-137 at the glutamatergic synapse. This figure summarizes the findings of Kwon et al. (2013), Zhao et al. (2013), Olde Loohuis et al. (2015), Siegert et al. (2015), and Thomas et al. (2017). miR-137 regulates presynaptic signaling by regulating vesicle trafficking in the axon terminal. miR-137 also targets the mRNA that encodes the L-type calcium channel subunit Cav1.2 (CACNA1C), which has also been linked to schizophrenia with genome-wide significance. Postsynaptically, miR-137 regulates the levels of glutamatergic receptor subunits GluA1 (GRIA1) and GluN2A (GRIN2A), and bioinformatic predictions suggest that miR-137 may target the metabotropic glutamate receptor mGluR5 (GRM5) as well as ErbB4, which regulates the strength of glutamatergic synapses. mGluR5 signaling, in turn, increases miR-137 levels. miR-137 also regulates proteins within the PI3K-Akt-mTOR pathway, e.g., p55g, to regulate neuronal responses to BDNF and Nrg1 signaling. miR-137 may regulate PI3K-Akt-mTOR signaling downstream of mGluR receptors as well. Figure from Thomas et al. (2018), obtained under a creative commons license 3 and 4.

These studies illustrate the ability of miRNAs to affect the human brain by shaping neuronal communication and offer a mechanism by which miRNA deregulation may contribute to the development of psychiatric disorders (Figure 15).

Abnormal expression levels of miRNAs, including miR-132, miR-23a, miR-93 and miRNA-148b upregulation and miR-106b, miR-146b downregulation, were observed in the cerebellar cortex of autistic patients (Abu-Elneel et al., 2008). Similar dysregulation of miRNA expression was also observed in serum and lymphoblastoid cells of autistic patients (Talebizadeh et al., 2008; Sarachana et al., 2010; Mundalil Vasu et al., 2014), while increased levels of miR134-5p and miR138-5p were also reported (Hirsch et al., 2018).

In the SFARI database^2^, miR-137 also is one of the 202 genes deemed to present suggestive evidence of predisposition to autism and shows clear genetic association with ASD (Mahmoudi and Cairns, 2017). Individuals with ASD (Carter et al., 2011), intellectual disability (Willemsen et al., 2011), and syndromic obesity (D’Angelo et al., 2015; Tucci et al., 2016) had microdeletions on chromosome 1 at position p21.3, impacting MIR137. When several genome-wide association studies were combined, meta-analyses revealed that MIR137 was strongly related with schizophrenia. In the region 3.6 kb upstream of MIR137 (1:g.98515539A > T), an uncommon functional enhancer variant was discovered to be connected to schizophrenia and bipolar disorder (Duan et al., 2014). In heterozygous conditional-knockout mice, partial loss of MIR137 led to dysregulated synaptic plasticity, repetitive behavior, and decreased learning and social behavior; treatment with papaverine, an inhibitor of PDE10A, or PDE10A knockdown alleviated these impairments (Cheng et al., 2018).

## 4. Conclusion

In our previous work (Beopoulos et al., 2022), we had explored the possible impacts of increased placental release of serotonin, subsequent to sustained maternal low-grade inflammation in early to mid-pregnancy, upon the probability of ASD pathogenesis in the offspring and found strongly corroborating evidence in a multiplicity of reports. Hence, although subject to numerous caveats involving fetal genotypes and environmental factors affecting maternal wellbeing, maternal low-grade systemic inflammation, reported be about 25% in a sample of adult US women (Ford et al., 2004), particularly during early and mid-pregnancy, seems to be a major determinant for significantly increased risk of ASD pathogenesis.

The increased 5-HT supply to the growing fetus cannot, however, be the only inflammatory-associated component that is implicated in the pathophysiology of ASD. Indeed, a number of additional inflammation-related factors, including interferons, other interleukins, TGFβ, TNFα and β, etc., can incorporate the fetal blood through the placenta and have been proven to have a major impact on *in utero* fetal brain development (Wei et al., 2013; Theoharides et al., 2016; Tsivion-Visbord et al., 2020). Additionally, a comparison of results from several studies reveals that the type of causal immunogen, the gestational stage at which the maternal inflammatory reaction starts, its severity, and its duration might all have an impact on how maternal inflammation affects neurodevelopment [for example, see (Boksa, 2010)]. The inflammatory response of the placenta during pregnancy can have sexually dimorphic consequences on fetal development (Clifton, 2005; Mueller and Bale, 2008), and the postpartum increase in neonatal testosterone in males can affect long-term CNS function (Forgie and Stewart, 1993; Wilson and Davies, 2007). This led us to postulate that in the pathogenesis of ASD, RNA epitranscriptomic dysregulations might probably take precedence over differential epigenetic (methylation/acetylation) modifications and to investigate the dynamic mechanisms most likely to be affected.

The link between maternal inflammation/immune activation, RNA epitranscriptomics and neurodevelopment has been demonstrated in studies in which mice were treated prenatally with the inflammatory mediator poly (I:C) or LPS on day 9 of gestation (E9). Offspring treated prenatally with LPS showed increased neuronal density at postnatal day (PD)14 but no changes were observed in arborization mediators such as reelin. However, prenatal poly(I:C)-treated mice had fewer reelin-positive cells in the dorsal stratum oriens at PD28, whereas increased expression of GAD67 (catalyzes GABA production from L-glutamic acid) in the ventral stratum oriens was observed in prenatal LPS-treated male mice and prenatal poly(I:C) during the prenatal period (Harvey and Boksa, 2012).

In another study, the offspring of mice treated with poly (I:C) at E9 showed marked behavioral deficits at PD24 with the brains of the fetuses increasing A-to-I RNA editing levels, independently of mRNA expression levels, affecting genetic pathways related to brain development (Tsivion-Visbord et al., 2020). DACT3, COG3, and GRIA2 were amongst the mRNAs presenting the greatest increases in A-to-I editing. Dact3 plays a crucial role in the mouse central nervous system’s embryonic development (Fisher et al., 2006), and COG3, a component of the COG complex, interacts with BLOC-1, which, along with the AP-3 complex, is necessary to direct membrane protein cargos into vesicles assembled at cell bodies for delivery into neurites and nerve terminals (Lee et al., 2012). Neurite extension is another function of the BLOC-1 complex that relies on its association with SNARE proteins (Setty et al., 2007). Although there are intriguing mechanistic differences between rodent and the corresponding human regional brain development patterns (Lim and Alvarez-Buylla, 2016), the above reports clearly establish mechanistic links between maternal immune activation during early pregnancy and brain development alterations together with long-term behavioral deficits in the offspring.

According to the present work, the neuroanatomic characteristics of ASD are found to be better explained through dysregulated RNA epitranscriptomic mechanisms occurring early during brain development than through differential epigenetic markings alone. Among such mechanisms, dysregulated mRNA-specific poly(A)-tail modulation is strongly correlated with genetic factors and high-risk genes associated with ASD pathogenesis; alterations in mRNA alternative splicing concurrently with dysregulated A-to-I RNA editing, correlates with the functional characteristics of neuronal receptors and channels organizations. The latter primarily influences GABAA and serotonin 2C receptors, glutamate and voltage-gated ion channels, filamins and actin organization, and eventually synaptic structures and plasticity. A-to-I RNA editing also induces changes in mi/siRNA profiling that impact neuronal organization, dendritic spine density and synaptic transmission by changing the interaction affinities with binding partners while also affecting neuronal migration, glial differentiation and regional brain patterning. RNA epitranscriptomics may well account for the enormous genetic and symptomatic heterogeneities that are systematically associated with psychiatric disorders at large and not with ASD only (Gandal et al., 2018; Walker et al., 2019).

## Data availability statement

The original contributions presented in this study are included in this article/supplementary material, further inquiries can be directed to the corresponding author.

## Author contributions

FI: conceptualization and methodology. MG: software. FI, AB, MG, and AF: validation and resources. FI and AB: formal analysis, data curation, and writing—original draft preparation. FI, AB, and AF: writing—review and editing. MG: project administration. All authors have read and agreed to the published version of the manuscript.

## Footnotes

1
https://gene.sfari.org/database/gene-scoring/

2
https://gene.sfari.org
